# The histone H3-lysine 4-methyltransferase Mll4 regulates the development of growth hormone-releasing hormone-producing neurons in the mouse hypothalamus

**DOI:** 10.1038/s41467-020-20511-7

**Published:** 2021-01-11

**Authors:** Christian Huisman, Young A. Kim, Shin Jeon, Bongjin Shin, Jeonghoon Choi, Su Jeong Lim, Sung Min Youn, Younjung Park, Medha K. C., Sangsoo Kim, Soo-Kyung Lee, Seunghee Lee, Jae W. Lee

**Affiliations:** 1 Department of Pediatrics, Oregon Health and Science University, Portland, OR USA; 2 College of Pharmacy and Research Institute of Pharmaceutical Sciences, Seoul National University, Seoul, Korea; 3 Department of Biological Sciences, University at Buffalo, Buffalo, NY 142604 USA; 4 Department of Bioinformatics and Life Science, Soongsil University, Seoul, Korea

**Keywords:** Disease model, Neurogenesis, Neuronal development

## Abstract

In humans, inactivating mutations in *MLL4*, which encodes a histone H3-lysine 4-methyltransferase, lead to Kabuki syndrome (KS). While dwarfism is a cardinal feature of KS, the underlying etiology remains unclear. Here we report that Mll4 regulates the development of growth hormone-releasing hormone (GHRH)-producing neurons in the mouse hypothalamus. Our two *Mll4* mutant mouse models exhibit dwarfism phenotype and impairment of the developmental programs for GHRH-neurons. Our ChIP-seq analysis reveals that, in the developing mouse hypothalamus, Mll4 interacts with the transcription factor Nrf1 to trigger the expression of GHRH-neuronal genes. Interestingly, the deficiency of *Mll4* results in a marked reduction of histone marks of active transcription, while treatment with the histone deacetylase inhibitor AR-42 rescues the histone mark signature and restores GHRH-neuronal production in *Mll4* mutant mice. Our results suggest that the developmental dysregulation of Mll4-directed epigenetic control of transcription plays a role in the development of GHRH-neurons and dwarfism phenotype in mice.

## Abstract (web summary)

Mutations in the MLL4 gene can cause Kabuki syndrome, whose underlying molecular mechanisms are unclear. Here, the authors show that Mll4 epigenetically regulates the transcriptional program leading to the formation of GHRH-neurons in the developing mouse hypothalamus.

## Introduction

Kabuki syndrome (KS), caused by haploinsufficiency of *MLL4* or *UTX*, is a human developmental disorder that affects multiple tissues. One of the consistent cardinal features of KS is stunted growth and postnatal short stature^1^. For instance, a report on growth data in 39 KS patients revealed that postnatal growth retardation is a clinical feature in all cases^2^. To date, however, the molecular etiology underlying stunted growth and short stature in KS patients remains ambiguous. The postnatal growth is controlled by the hypothalamus–pituitary gland–liver axis^3^. First, growth hormone-releasing hormone (GHRH)-neurons in the hypothalamus release GHRH, which then stimulates secretion of the growth hormone (GH) from the pituitary gland. GH, in turn, induces the expression of insulin-like growth factor 1 (IGF1) in the liver, which controls bone epiphyses, muscle and adipose tissue development, growth plates development, and glucose homeostasis^4^. It is noteworthy that all 18 genetically confirmed prepubertal KS children successfully underwent catch-up growth after receiving a year-long treatment with recombinant human growth hormone (GH)^5^. These results suggest that the deficits of human KS patients in GH signaling should involve the production of GHRH (in the hypothalamus) and/or GH (in the pituitary) but not the events downstream of the pituitary in GH signaling pathway.

GHRH neurons are located in the hypothalamic arcuate nucleus (ARC, aka ARH), which centrally regulates diverse homeostatic processes critical for survival and reproduction^3,6–8^. The single-cell RNA-seq (scRNA-seq) analyses of the adult mouse ARC uncovered that the ARC contains as many as 24 distinct neuronal type^9^. Among these ARC neuronal types, GHRH-neurons constitute the hypothalamus–pituitary–liver regulatory axis, which directs linear growth. AgRP-neurons enhance food intake and reduce energy expenditure by releasing the neuropeptides NPY and AgRP, while POMC-neurons perform the opposite actions using the neuropeptides αMSH (cleaved from POMC) and CART^6^. Kiss1-neurons control reproduction via the *Kiss1*-encoded neuropeptide Kisspeptin, which triggers the secretion of gonadotrophin-releasing hormone (GnRH) from the hypothalamic GnRH-neurons^8^. To understand the developmental process for the ARC, we recently established gene expression profiles for each ARC neuronal type undergoing the cell fate specification and differentiation in the mouse embryonic hypothalamus and identified 83 genes that are most specifically enriched in developing GHRH-neurons relative to other developing ARC neurons^10^.

The genetic cause of KS has been relatively well understood. A majority of KS is caused by mutations in the *MLL4* gene, but some cases of KS are due to mutations in the *UTX* gene^1^. The human genetics of KS corroborate the finding that MLL4 and UTX together form a histone-modifying enzyme complex named MLL4-complex, which controls gene expression, often in cell type-specific manners^11,12^. These data also strongly support the notion that the defect in MLL4/UTX-directed epigenetic gene regulation is a main contributing factor to human KS phenotypes. MLL4 is a histone H3-lysine 4 (H3K4)-methyltransferase. The methylation patterns in the histone H3K4 residue form versatile epigenetic marks that are intimately linked to the induction of associated genes. The promoters are decorated by H3K4 tri‐methylation (H3K4me3), while the enhancers are marked by H3K4 mono- and di‐methylations (H3K4me1 and H3K4me2)^13^. In lower eukaryotes, a singular H3K4‐methyltransferase Set1 performs H3K4 methylations through a large steady‐state Set1‐complex^13^. The H3K4‐methyltransferase action is divided among multiple complexes in higher eukaryotes. *Drosophila* has dSet1-, Trx-, and Trr-complexes that generate H3K4-methylation marks^14^. Mammals have six similar complexes, collectively named Set1-like complexes, and each complex contains one of the six H3K4‐methyltransferase, Set1α or its paralog Set1β, MLL1 or its paralog MLL2 (aka WBP7), or MLL3 (aka KMT2C) or its paralog MLL4 (aka KMT2D)^11,12^. We have identified the first two mammalian Set1‐like complexes, MLL3- and MLL4-complexes^15,16^. Interestingly, among the six mammalian Set1-like complexes, only the MLL3- and MLL4-complexes contain the H3K27‐demethylase UTX^11^, which removes the transcriptionally repressive chromatin mark H3K27 methylation^13^. Thus, MLL3/4-complexes have two distinct enzymatic activities that lead to the open chromatin formation for transactivation of the target genes. Consistently, the MLL3/4-complexes function as transcriptional coactivators that trigger target gene expression, and the subunits in the MLL3/4-complexes serve as adaptors that recruit MLL3/4 and UTX to enhancers of their target genes by partnering with transcription factors bound to those enhancers^11,12^.

With regards to MLL3/4-directed epigenetic gene regulation, the interplay between the two histone marks H3K4-methyation and H3K27-modification is noteworthy. MLL3/4 have been shown to be the main enzymes that generate the enhancer marks H3K4me1 and H3K4me2 (refs. ^17–21^). H3K4me1/2 marks concomitantly occur with another active enhancer mark H3K27ac, often preceding H3K27ac^22,23^. Several studies provide the molecular basis for the co-occurrences of H3K4me1/2 and H3K27ac marks^23–26^ and suggest that MLL3/4-directed H3K4me1/2 are intimately linked to H3K27ac.

In this paper, we report that Mll4 governs the development of GHRH-neurons in the mouse hypothalamus via its epigenetic regulatory activity. Further, we found that Mll4 triggers the expression of GHRH-neuronal genes during development, primarily by partnering with the transcription factor Nrf1 (for nuclear respiratory factor 1, aka α-Pal). Together, our study revealed that the deficiency of Mll4-directed epigenetic control of GHRH-neuronal genes results in a severe reduction of GHRH neurons and dwarfism in the mouse, providing crucial insights into the molecular etiology underlying dwarfism in human KS patients.

## Results

### *Mll4*^*+/−*^ mice exhibit dwarfism and the reduction of GHRH neurons

**Fig. 1:**
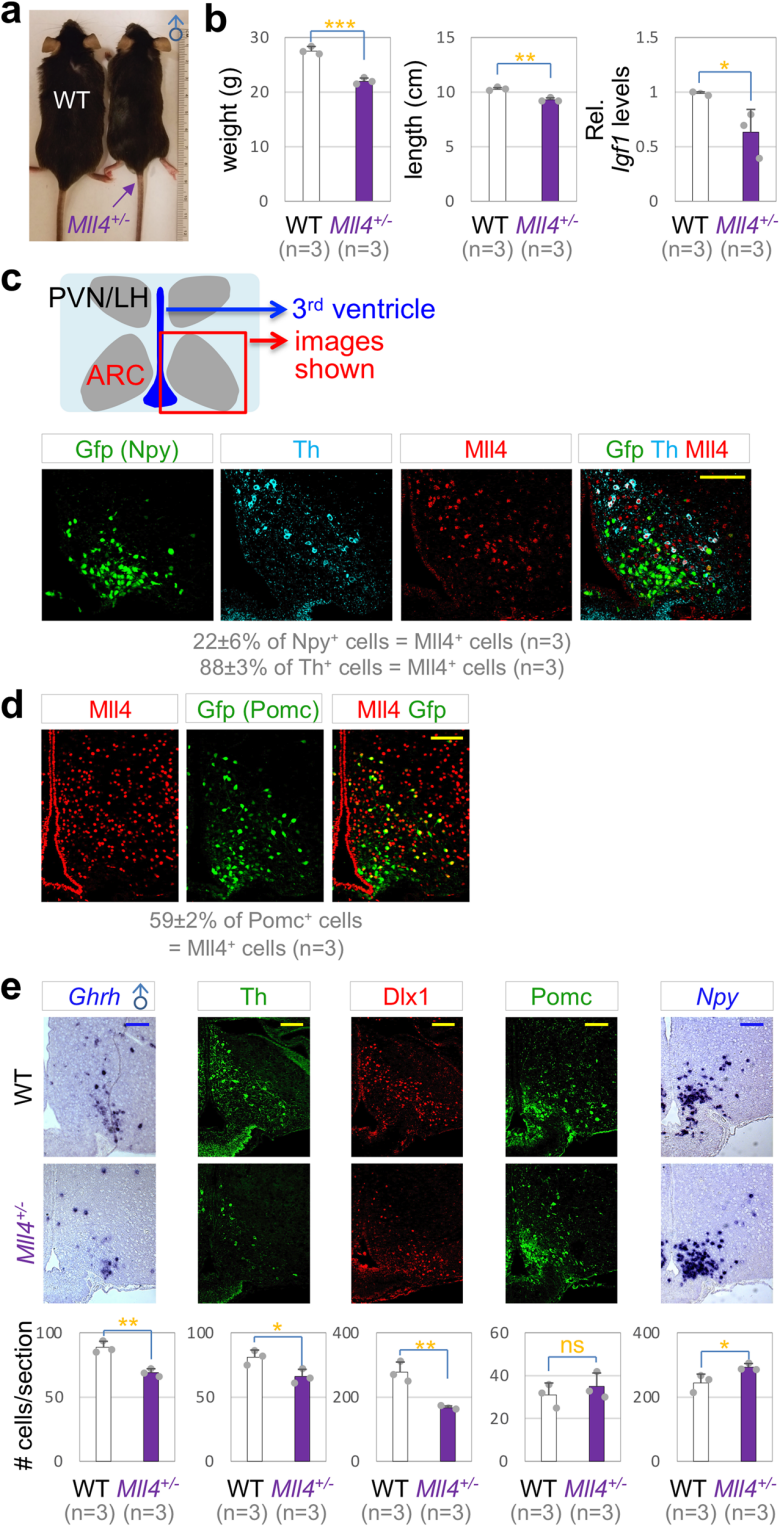
Dwarfism of *Mll4*^*+/−*^ mice. **a** *Mll4*^*+/−*^ male mice are smaller than their littermate WT mice at P80. **b** A significant reduction in body weight as well as linear length and liver *Igf1* levels in male *Mll4*^*+/−*^ mice relative to their littermate WT mice at P80. **c**, **e** Coexpression of Mll4 (IHC) with either Gfp driven by *Npy-Gfp* or Th (IHC) at P33 (**c**), and with Gfp driven by *Pomc-Gfp* at P33 (**d**). **e** Expression of *Ghrh*, Th, Dlx1, Pomc, and *Npy* in the ARC of male WT mice and their littermate *Mll4*^*+/−*^ mice at P80, assessed by ISH (*Ghrh, Npy*) and IHC (Th, Dlx1, POMC). Quantifications were done by counting the number of labeled cells in three rostral to caudal sections for each mouse, and the number of mice used is as indicated in parenthesis (**c**–**e**). The location of ISH/IHC images is schematically shown (**c**), which applies to all images in (**c**–**e**). Scale bars, 100 µm. Statistical differences were determined by two-sided Student’s *t*-test (**b**, **e**); *p < 0.05, **p < 0.01, ***p < 0.001, and not significant (ns). Column bars represent mean, error bars indicate the SD (**b**, **e**).

To test if *Mll4* haploinsufficiency leads to dwarfism in mice as it does in human, we monitored the growth of *Mll4*^*+/−*^ mice, in which a single *Mll4* allele is inactivated^27^. *Mll4*^*+/−*^ mice showed a significant reduction in body weight, linear length, and mRNA levels for *Igf1* in the liver (Fig. 1a, b, Supplementary Fig. 1a), indicating that *Mll4*^*+/−*^ mice have a deficit in GH signaling and faithfully recapitulate one of the cardinal features of KS, postnatal growth deficiency^1^.

To test the role of Mll4 in hypothalamic GHRH neurons, we monitored the expression pattern of Mll4 in the ARC. GHRH neurons are one of tyrosine hydroxylase (Th)^+^ neuronal types in the ARC^28^. Interestingly, Mll4 was expressed in ~88% of Th^+^ neurons that include GHRH neurons, but only in ~22% of Npy^+^ AgRP-neurons at postnatal day 33 (P33) in *Npy-Gfp* reporter mice that express Gfp in AgRP-neurons^29^ (Fig. 1c). In contrast, Mll4 was expressed in ~59% of *Pomc*^+^ cells in P65 *Pomc-eGfp* reporter mice^30^ (Fig. 1d). These results indicate that most GHRH-neurons express Mll4, whereas only a subset of AgRP- and POMC-neurons express Mll4.

Next, we tested if the number of GHRH-, AgRP-, and POMC-neurons changes in *Mll4*^*+/−*^ mice using a panel of markers. GHRH-neurons significantly reduced, as determined by GHRH-neuronal markers, *Ghrh*, Th, and Dlx1, in *Mll4*^*+/−*^ mice relative to littermate WT control mice (Fig. 1e). In contrast, the number of POMC-neurons did not change and AgRP-neurons mildly increased in *Mll4*^*+/−*^ mice (Fig. 1e). Our data indicate that the generation or survival of GHRH-neurons is impaired in *Mll4*^*+/−*^ mice, and also suggest that the deficiency of GHRH-neurons is likely a strong contributing factor to the dwarfism in *Mll4* haploinsufficiency.

### The inactivation of *Mll4* in the developing hypothalamus leads to a drastic reduction of GHRH neurons and dwarfism

**Fig. 2:**
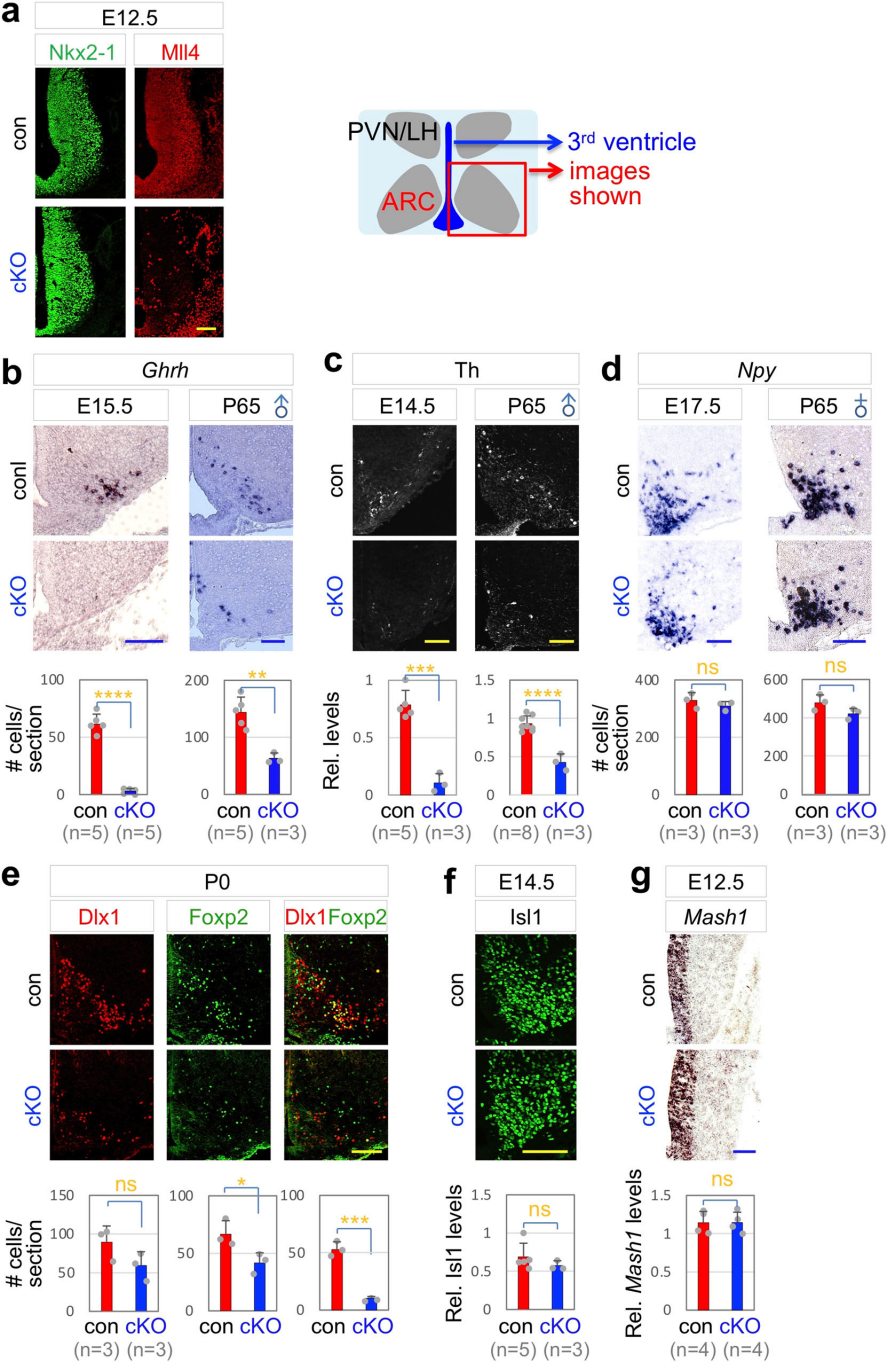
Ghrh expression impaired in *Mll4*-cKO mice. **a** E12.5 *Mll4*-cKO embryos show a drastic reduction in Mll4 expression in the developing hypothalamus. Similar results were obtained from at least three independent experiments. **b**–**d** Expression of *Ghrh* (**b**) and Th (**c**), but not *Npy* (**d**), is significantly reduced in *Mll4*-cKO at both embryonic and adult stages. **e** Cells expressing Dlx1 or Foxp2 only as well as Dlx1/Foxp2 double-positive cells are reduced in P0 *Mll4*-cKO pups relative to their littermate P0 control pups. **f**, **g** Expression of Isl1 (**f**) and *Mash1* (**g**) was not significantly different between control embryos and their littermate *Mll4*-cKO embryos. The location of ISH/IHC images is schematically shown (**a**), which applies to all images in (**a**–**g**). Scale bars, 100 µm. Quantifications were done by counting the number of labeled cells or the relative intensity of signals in three rostral to caudal sections for each mouse. The number of mice used is as indicated below each genotype in parenthesis (**b**–**g**). Statistical differences were determined by two-sided Student’s *t*-test (**b**–**g**); *p < 0.05, **p < 0.01, ***p < 0.001, ****p < 0.0001, and not significant (ns). Column bars represent mean, error bars indicate the SD (**b**–**g**).

To test if Mll4 plays a cell-autonomous role in GHRH-neuronal development, we generated *Mll4* conditional knockout (*Mll4*-cKO) mice, in which *Mll4* was deleted in the embryonic ARC, by crossing *Mll4*^*f/f*^ and *Nkx2-1-Cre* mice^21,31^. In *Mll4*-cKO mice, Mll4 expression was largely eliminated in the developing ARC, but not in surrounding tissues, by embryonic day (E) 12.5 (Fig. 2a). At E14.5~E15.5, GHRH-neurons expressing *Ghrh* and Th were markedly reduced in *Mll4*-cKO mice compared to littermate control mice (Fig. 2b, c), indicating that the development of GHRH-neurons was impaired in the absence of Mll4. *Mll4*-cKO mice also showed a striking reduction of GHRH-neurons at P65 (Fig. 2b, c), indicating that the developmental defects of GHRH-neurons in *Mll4*-cKO embryos were not recovered at adult stage. In contrast to GHRH-neurons, the number of AgRP-neurons did not significantly change in *Mll4*-cKO mice (Fig. 2d). We also tested the marker for other hypothalamic neurons; *Trh*, a marker for the dorsomedial hypothalamus that does not express Nkx2-1^32^ and *Sf1*, a marker for a subregion of the ventromedial hypothalamus that expresses Nkx2-1^32,33^. Neither *Sf1*^*+*^ nor *Trh*^*+*^ cells were significantly altered in their numbers in *Mll4*-cKO mice (Supplementary Fig. 2), highlighting a relatively selective loss of GHRH-neurons in the *Mll4*-deficient hypothalamus.

To determine if the mere expression of *Ghrh* gene alone or the developmental program for GHRH-neurons was disrupted in *Mll4*-cKO mice, we examined the expression pattern of transcription factors that are crucial to drive GHRH-neuronal development. We have previously reported that Dlx1 and its paralog Dlx2 are required for the development of mouse Th^+^ neurons, which include GHRH-neurons^34^ and that Foxp2 is essential for the generation of mouse GHRH-neurons^10^. Mash1, expressed in neural progenitors in the ARC, and Isl1, expressed in ARC neurons, are involved in the development of multiple ARC neuronal types^35–37^. Interestingly, in *Mll4*-cKO mice, the number of cells co-expressing Dlx1 and Foxp2 was drastically reduced (Fig. 2e), but the levels of Isl1 and Mash1 did not significantly change (Fig. 2f, g). These results strongly suggest that the development of GHRH-neurons, rather than just the expression of *Ghrh*, is impaired in *Mll4*-cKO mice.

**Fig. 3:**
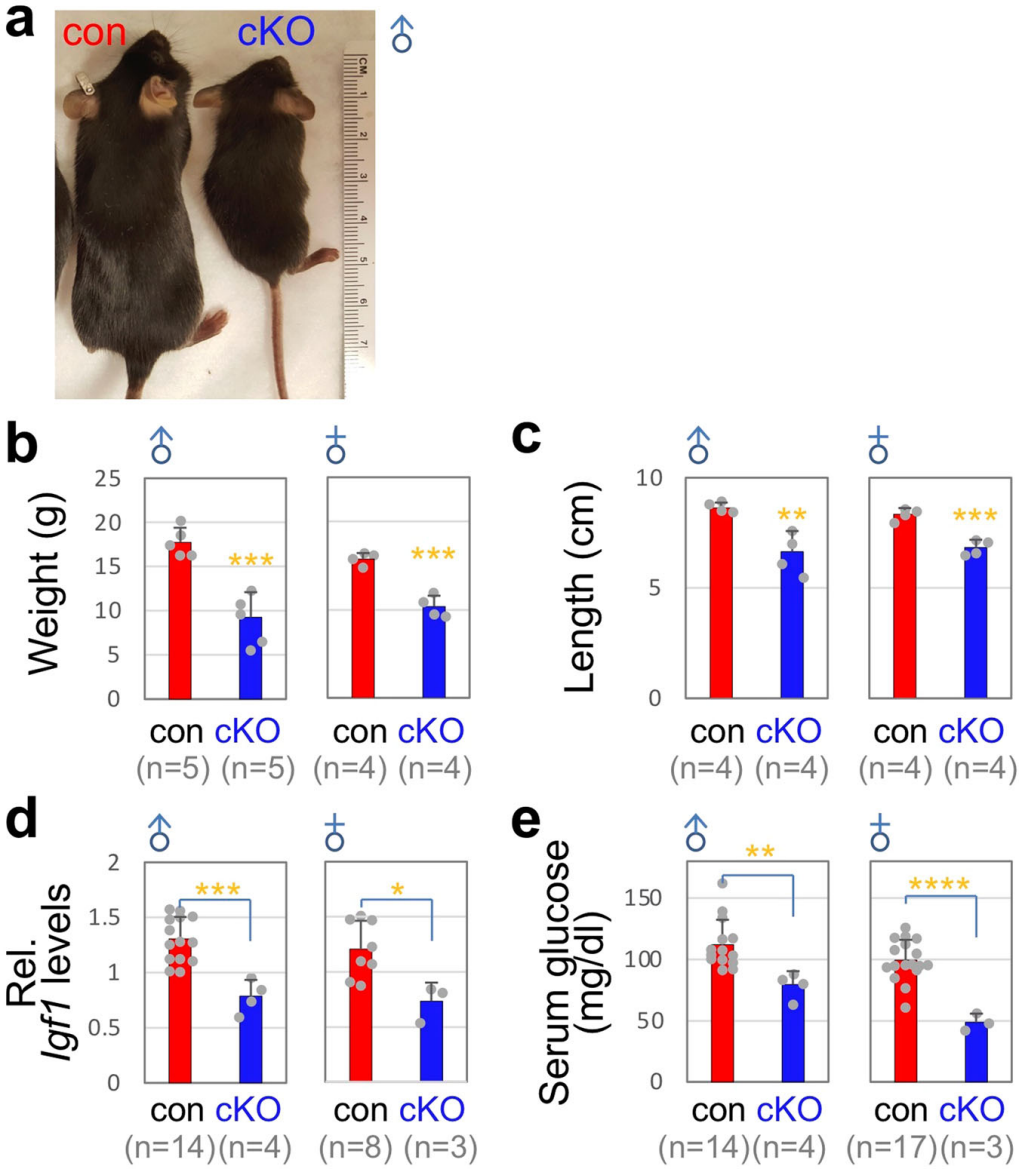
Dwarfism of *Mll4*-cKO mice. **a** *Mll4*-cKO mice are smaller than their littermate control mice (a representative image for males). **b**–**e** A significant reduction in body weight (**b**), linear length (**c**), liver *Igf1* mRNA levels (**d**), and serum glucose levels (**e**) in male and female *Mll4*-cKO mice relative to their littermate control mice at P40. The number of mice used is as indicated below each genotype in parenthesis (**b**–**e**). Statistical differences were determined by two-sided Student’s *t*-test; *p < 0.05, **p < 0.01, ***p < 0.001, and ****p < 0.0001. Column bars represent mean, error bars indicate the SD (**b**–**e**).

Next, we examined if *Mll4*-cKO mice exhibit any growth phenotypes, reflecting the developmental deficiency of GHRH-neurons. *Mll4*-cKO mice showed a marked reduction of both body weight and linear length (Fig. 3a–c). Furthermore, serum glucose levels and hepatic *Igf1* mRNA levels drastically decreased in *Mll4*-cKO mice (Fig. 3d, e), indicating deficits in the signaling of GH, downstream of GHRH. Milder but similar results were also obtained with *Mll4*-cHET (for conditional heterozygous knockout) mice (Supplementary Fig. 1b).

Together, our results establish that Mll4 is required for the proper generation of GHRH-neurons in the developing hypothalamus. Our data also clearly link the stunted growth of *Mll4* mutant mice to the impaired development of GHRH-neurons.

### Nrf1 is a major partner transcription factor of Mll4 in developing hypothalamus

**Fig. 4:**
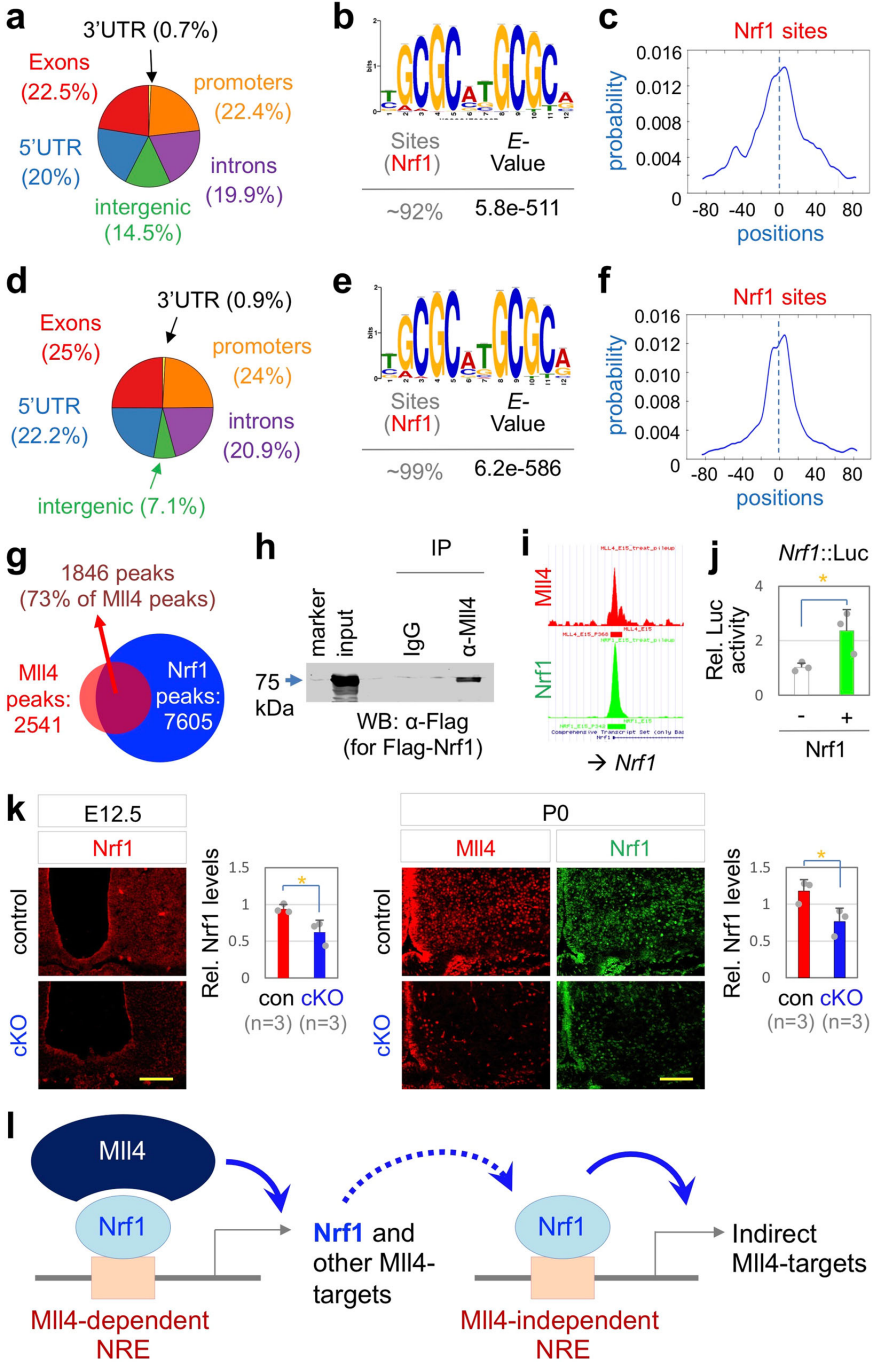
Nrf1 is a major partner transcription factor of Mll4 in developing hypothalamus. **a**–**f** Location of ChIP-seq peaks in E15 hypothalamus shown for Mll4 ChIP-seq (**a**) and Nrf1 Chip-seq (**d**), Nrf1-binding motif found in de novo motif analysis of the top 300 Mll4 peaks (**b**) and Nrf1 peaks (**e**), and the enrichment of the Nrf1-binding motif in the center of the peaks for Mll4 (**c**) and Nrf1 (**f**). **g** 73% of Mll4 ChIP-seq peaks overlap with Nrf1 ChIP-seq peaks, suggesting recruitment of Mll4 to a majority of its targets via Nrf1. **h** CoIP of Mll4 and Flag-Nrf1 in HEK293 cells. Similar results were obtained from at least three independent experiments. **i** An identical locus of the promoter region of *Nrf1* gene is associated with Mll4 and Nrf1 ChIP-seq peaks. **j** A luciferase reporter construct directed by the genomic region containing the *Nrf1*-associated Nrf1/Mll4-binding locus (**i**) is activated by ectopic expression of Nrf1 in HEK293 cells. Three independent experiments (each experiment done in duplicate) were analyzed together. **k** Nrf1 expression is reduced in *Mll4*-cKO at both E12.5 and P0 relative to their littermate controls (*n* = 3, each genotype). Scale bars, 100 µm. Statistical differences were determined by two-sided Student’s *t*-test (**j**, **k**); *p < 0.05. Column bars represent mean, error bars indicate the SEM (**j**) and SD (**k**). **l** A model for direct and indirect target genes of Mll4 in the developing hypothalamus. The *Nrf1* gene is a direct target of Mll4:Nrf1, and therefore genes regulated by Nrf1 alone can be classified as indirect target genes of Mll4.

To determine the molecular mechanism by which Mll4 directs the development of GHRH-neurons, we defined genome-wide Mll4-binding loci in the developing hypothalamus by performing ChIP-seq in the mouse hypothalamus at E15, a time point when GHRH-neurons are being actively specified, with the ChIP-seq quality Mll4 antibody that we developed^27^. Our ChIP-seq analyses identified 2541 Mll4-bound ChIP-seq peaks (*p* < 0.001, FDR < 10%). Interestingly, ~85.5% of Mll4 ChIP-seq peaks were located in the promoters and gene bodies and only ~14.5% of Mll4-bound peaks were found in the intragenic regions in the developing hypothalamus (Fig. 4a). This Mll4-occupany pattern differs from the previous finding that Mll4 is mainly enriched in the intergenic enhancer regions in the adipocytes^21^.

To identify partner transcription factors that mediate the recruitment of Mll4 to its target genomic loci in the developing hypothalamus, we performed de novo motif analyses on the top 300 Mll4 ChIP-seq peaks using the algorithms MEME, DREME, and TOMTOM^38–40^. Intriguingly, a single motif, which resembles the consensus binding site for the transcription factor Nrf1 (ref. ^41^), was found in ~92% of the top 300 Mll4 ChIP-seq peaks (Fig. 4b). The Nrf1 motif was enriched in the summit of Mll4 ChIP-seq peaks (Fig. 4c). This genome-wide analyses indicate that Nrf1 is primarily responsible for recruiting Mll4 to most Mll4-target loci in E15 developing hypothalamus.

To further test the role of Nrf1 in Mll4-directed gene regulation in the developing hypothalamus, we performed ChIP-seq in E15 hypothalamus using ChIP-seq quality Nrf1 antibody that we generated, and identified 7605 Nrf1-bound genomic regions in the developing hypothalamus (*p* < 0.001, FDR < 10%). Similar to Mll4, Nrf1 primarily occupied the promoters and gene bodies (Fig. 4d). De novo motif analyses uncovered that ~99% of Nrf1-bound genomic regions contain the Nrf1 motif (Fig. 4e). Validating that the Nrf1 motif is the binding site for Nrf1, the motif was enriched in the summit of Nrf1 ChIP-seq peaks (Fig. 4f). The direct comparison of genome-wide binding patterns of Mll4 and Nrf1 revealed that 73% of Mll4 ChIP-seq peaks are also the binding sites for Nrf1 (Fig. 4g). These data suggest that Nrf1 mediates the recruitment of Mll4 to a majority of Mll4-target genes in the developing hypothalamus (Fig. 4g). Further supporting this notion, Mll4 associated with Nrf1 in HEK293 cells, as determined by co-immunoprecipitation (coIP) assays (Fig. 4h).

Notably, Mll4 and Nrf1 co-occupied the promoter of the *Nrf1* gene itself (Fig. 4i), raising the possibility that Nrf1 auto-regulates its own transcription by recruiting Mll4 as a transcriptional coactivator. Indeed, the ectopic expression of Nrf1 enhanced the transcriptional activity of the Nrf1/Mll4-binding region in the *Nrf1* gene in HEK293 cells, as monitored using luciferase reporter assays (Fig. 4j). The expression of Nrf1 was severely reduced in the ARC region of *Mll4*-cKO mice relative to their littermate controls at E12.5 and P0 (Fig. 4k), supporting the notion that Mll4 induces the expression of Nrf1 by binding the *Nrf1* gene in the developing hypothalamus (Fig. 4i).

Together, these results suggest that, in the developing hypothalamus, Mll4 employs Nrf1 as the major partner transcription factor to bind and induce its ‘direct’ target genes. Given that Mll4 activates the expression of Nrf1, Mll4 is also expected to positively regulate the genes bound by Nrf1 alone without Mll4, which represent ‘indirect’ target genes of Mll4 (Fig. 4l).

### Mll4 positively regulates GHRH-neuronal genes in the developing hypothalamus

**Fig. 5:**
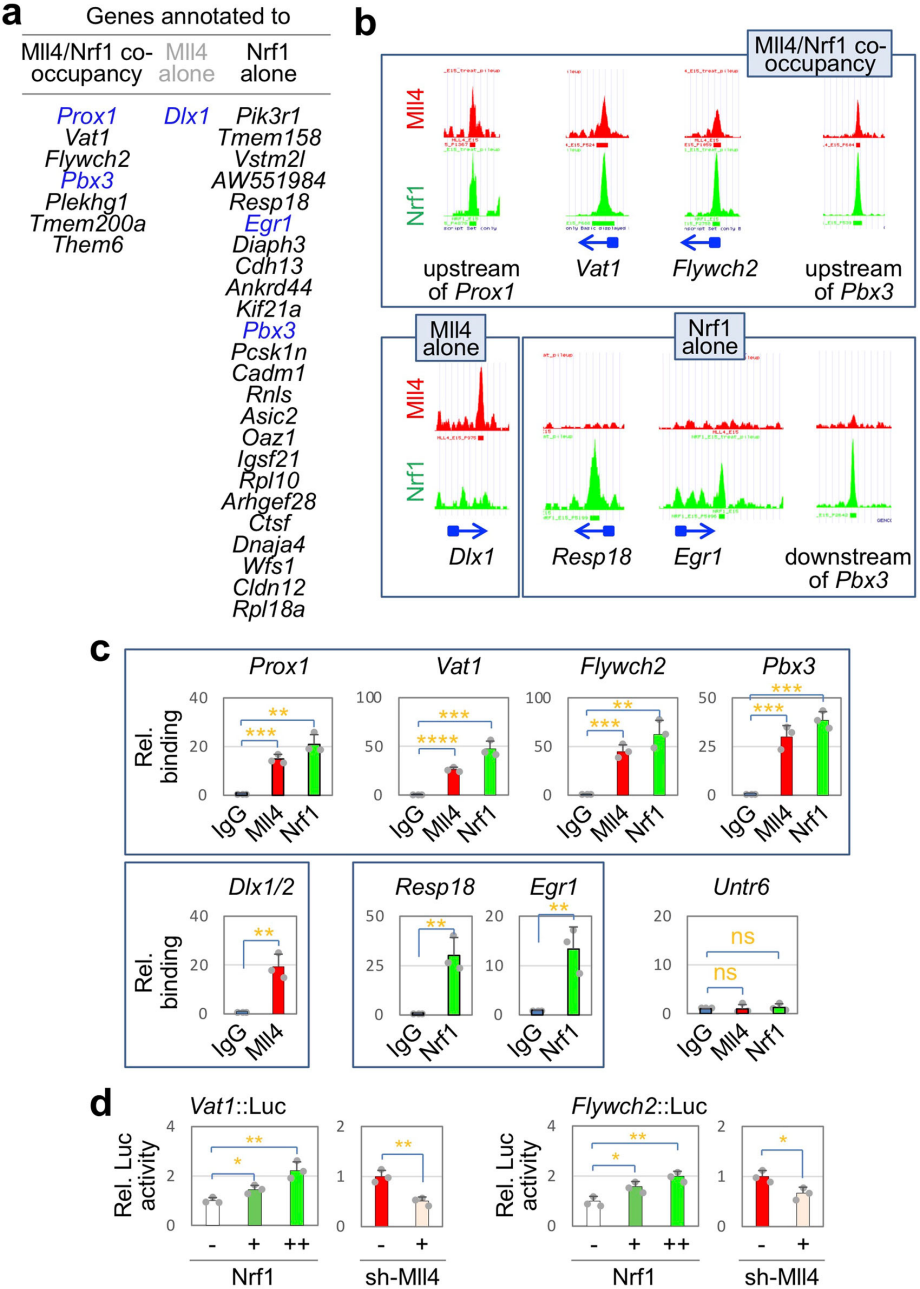
Target genes of Mll4 in developing mouse GHRH neurons. **a** List of genes in developing mouse GHRH-neurons, which are associated with either Mll4 or Nrf1 ChIP-seq peaks or both Mll4 and Nrf1 ChIP-seq peaks. Genes encoding transcription factors are highlighted in blue. **b** Mll4 and Nrf1 ChIP-seq peaks for representative target genes of Mll4 in developing GHRH-neurons. The location of each peak with regard to its associated gene is marked by an arrow for the direction of the gene and a square for the 5′ UTR (both in blue). **c** Independent validation ChIP experiments with E15 hypothalamus and IgG (controls) and antibodies against Mll4 and Nrf1 reveal recruitment of Mll4 and Nrf1 to representative target genes of Mll4. Column bars represent mean of experiments with three independent embryos, error bars indicate the SD. **d** Luciferase reporter assays reveal that the common ChIP-seq peak areas for Mll4 and Nrf1 in *Vat1* and *Flywch2* are responsive to ectopic expression of Nrf1 and shRNA against Mll4. Column bars represent mean of three independent experiments, error bars indicate the SEM. Statistical differences were determined by two-sided Student’s *t*-test; not significant (ns), *p < 0.05, **p < 0.01, ***p < 0.001, and ****p < 0.0001.

To identify Mll4/Nrf1-target genes that are important in GHRH-neuronal development, we integrated Mll4 and Nrf1 ChIP-seq datasets with our scRNA-seq analyses of E15 ARC neurons, which revealed 83 genes that are most specifically enriched in developing GHRH-neurons relative to other ARC neuronal types^10^. Among the 83 genes, 7 genes recruit both Mll4 and Nrf1 to the same loci (Fig. 5a, b, Supplementary Fig. 3), suggesting that Nrf1 mobilizes Mll4 to these 7 genes thereby activating their expression (Fig. 4l). These Nrf1-Mll4-co-occupancy genes include two transcription factor genes *Prox1* and *Pbx3* and five non-transcription factor genes, *Vat1*, *Flywch2*, *Plekhg1*, *Tmem200a*, and *Them6* (Fig. 5a, b, Supplementary Fig. 3). Among the 83 genes, 24 genes including the transcription factor gene *Egr1*, are associated with the genomic regions that recruit only Nrf1, not Mll4, thus representing potential ‘indirect’ Mll4-target genes (Fig. 4l). Interestingly, Mll4 binds to the promoter/5′UTR of the *Dlx1* gene, but Nrf1 does not (Fig. 5a, b), suggesting that Mll4 induces Dlx1 expression in the developing hypothalamus by collaborating with other transcription factor. Overall, our systematic bioinformatic analyses revealed several ‘direct’ and ‘indirect’ target genes of Mll4 in the developing GHRH-neurons. Notably, these Mll4-target genes include Dlx1, Prox1, Egr1, and Pbx3, which have been shown or proposed to play a role in the development of GHRH-neurons^10,34^.

To validate the ChIP-seq data, we performed independent ChIP assays in E15 hypothalamus using antibodies against Mll4 and Nrf1. Consistent with ChIP-seq data, both Mll4 and Nrf1 were recruited to the common ChIP-seq peak regions for Mll4 and Nrf1 regions annotated to *Prox1*, *Vat1*, *Flywch2*, and *Pbx3*, but not to the negative control genomic region *Untr6* (for *Untr*anslated region on chromosome *6*)^42^ (Fig. 5c). Also, Nrf1 bound to ChIP-seq peak regions in *Resp18* and *Egr1*, and Mll4 bound to *Dlx1* (Fig. 5c).

To test if Nrf1/Mll4-bound genomic genes act as enhancers responding to Nrf1 and Mll4, we generated the luciferase reporters linked to Nrf1-Mll4-co-occupancy regions annotated to *Vat1* and *Flywch2*, named *Vat1::Luc* and *Flywch2::Luc*. Both luciferase reporters were activated by Nrf1 in a dose-dependent manner and suppressed by Mll4 knockdown with shRNA against Mll4 in HEK293 cells (Fig. 5d), suggesting that Nrf1 and Mll4 enhance the transcription activity of their binding regions in cells.

**Fig. 6:**
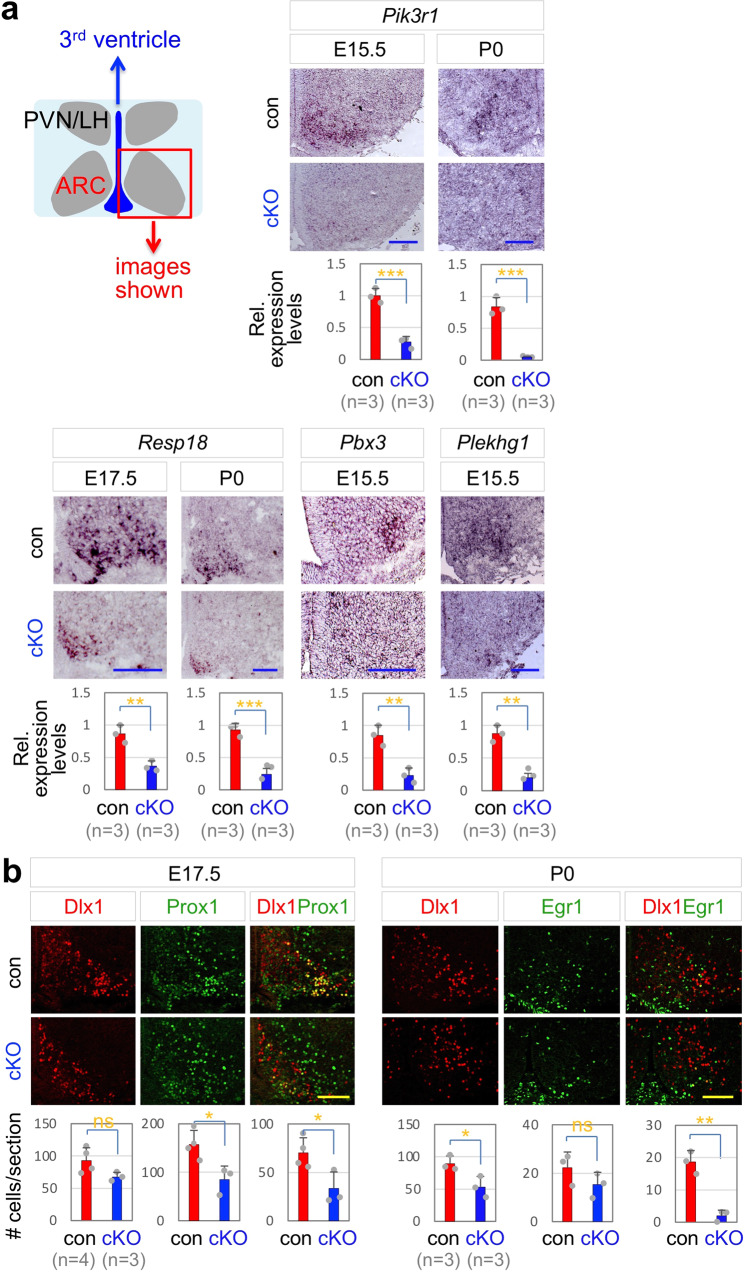
*Mll4*-cKO show reduced expression of Mll4-target genes in developing GHRH-neurons. **a** ISH analyses of expression of Mll4-target genes in *Mll4*-cKO and their littermate controls at indicated stages. **b** IHC analyses of coexpression of transcription factors whose genes are targeted by Mll4; the number of Dlx1 only, Prox1 only, and Dlx1/Prox1 double-positive cells at E17.5, and the number of Dlx1 only, Egr1 only, and Dlx1/Egr1 double-positive cells at P0 in *Mll4*-cKO and controls. The number of mice used is as indicated below each genotype in parenthesis (**a**, **b**). The location of ISH/IHC images is schematically shown (**a**), which applies to all images in (**a**, **b**). Statistical differences were determined by two-sided Student’s *t*-test; *p < 0.05, **p < 0.01, and ***p < 0.001. Column bars represent mean, error bars indicate the SD. Scale bars, 100 µm.

We predicted that the expression of the direct and indirect Mll4-target genes in mouse developing GHRH-neurons would be downregulated in the absence of Mll4. To test this idea, we examined the expression levels of potential Mll4-target genes in the ARC of *Mll4*-cKO mice using in situ hybridization (ISH) and immunohistochemistry (IHC) analyses. *Mll4*-cKO mice showed significant downregulation of all tested target genes, such as *Pbx3* and *Plekhg1*, which are annotated to Nrf1-Mll4-co-occupancy regions (i.e., direct Mll4-target genes), and *Pik3r1* and *Resp18*, which are associated with Nrf1 peak but not Mll4 peak (i.e., indirect Mll4-target genes) (Fig. 6a). While Dlx1, Prox1, and Egr1 are highly and significantly enriched in developing GHRH-neurons, they are also expressed in other ARC neuronal types, such as the expression of Dlx1 in developing n8/n9 TH- neurons^10^. Thus, to monitor the expression of Dlx1, Prox1, and Egr1 in developing GHRH-neurons, we performed the double immunofluorescence staining with a combination of antibodies against Dlx1 and Prox1 or Egr1. Dlx1/Prox1- and Dlx1/Egr1 double-positive cells in the ARC were markedly reduced in the ARC of *Mll4*-cKO mice relative to their littermate controls (Fig. 6b), indicating the downregulation of these transcription factors in GHRH-neuronal lineage and the impaired transcription program directing the development of GHRH-neurons.

Together, these studies strongly suggest that Mll4 governs GHRH-neuronal development by inducing transcription program crucial for GHRH-neuronal differentiation and also by upregulating non-transcription factor genes that are relatively specifically enriched in developing GHRH-neurons.

### Mll4 directs GHRH-neuronal development via its epigenetic regulatory activity

**Fig. 7:**
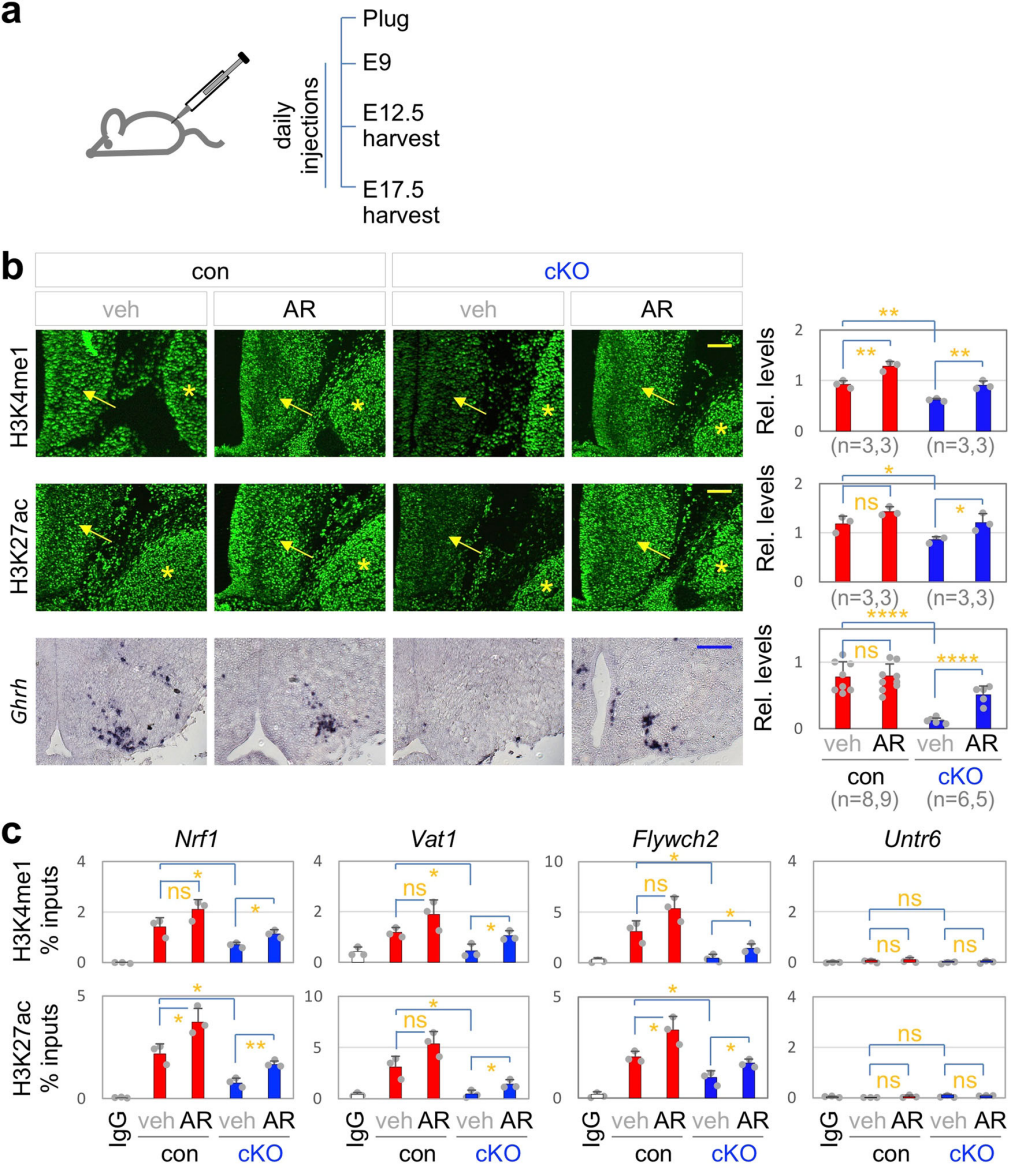
AR-42 restores *Ghrh* expression in *Mll4*-cKO mice. **a** Schematics for AR-42 experiments. From 9 days after a plug was observed, vehicle or AR-42 was injected to pregnant dams daily, followed by embryo harvests at E12.5 or E17.5. **b** IHC analyses of relative levels of H3K4me1 and H3K27ac in embryos harvested at E12.5 as well as ISH analyses of *Ghrh* expression in embryos harvested at E17.5. Quantification of H3K4me1/H3K27ac levels in the developing ARC (arrows) done relative to the signals in the adjacent tissues (the trigeminal ganglion, indicated by asterisks). The number of mice used is as indicated below each genotype in parenthesis. Scale bars, 100 µm. **c** Embryos harvested at E15 (*n* = 3, each genotype and condition) were subject to ChIP analyses with IgG (controls) or antibodies against H3K4me1 or H3K27ac, followed by qPCR quantification of H3K4me1/H3K27ac levels on genomic region containing the Mll4/Nrf1 ChIP-seq peaks associated with *Nrf1*, *Vat1*, *Flywch2*, and *Untr6* (negative control locus). Statistical differences were determined by two-sided Student’s *t*-test (**b**, **c**); *p < 0.05, **p < 0.01, ****p < 0.0001, and not significant (ns). Column bars represent mean, error bars indicate the SD (**b**, **c**).

Next, to ask if Mll4 triggers the transcriptional activation of its target genes in the GHRH-neuronal lineage via its epigenetic regulatory activity that induces transcriptionally active chromatin landscape, we sought for the method to trigger transcriptionally active chromatin, marked by H3K27ac, in *Mll4*-deficient mice. Given that the HDAC inhibitor AR-42 restored the active chromatin in the hippocampus and also rescued the hippocampal memory defects in *Mll4*^*+/−*^ mice^43^, we employed AR-42. We performed daily intraperitoneal injection of 50 mg/kg of AR-42 into pregnant dams from 9 days after a vaginal plug detection until harvesting the embryos (Fig. 7a). We first monitored the active chromatin marks at E12.5. H3K4me1 and H3K4me2 levels substantially reduced in the developing ARC, but not in the surrounding tissues, in *Mll4*-cKO embryos, whereas H3K4me3 levels did not show any change (Fig. 7b, Supplementary Fig. 4a). These data suggest that Mll4 is the main enzyme generating H3K4me1 and H3K4me2 marks in the developing ARC. Interestingly, H3K27ac levels also significantly deceased in *Mll4*-deficient ARC (Fig. 7b), consistent with the notion that enhancer marks H3K4me1/2 and H3K27ac occur concomitantly^22,23^. AR-42 treatment restored both H3K4me1/2 and H3K27ac levels in E12.5 Mll4-cKO embryos (Fig. 7b). To monitor the epigenetic modification in individual Mll4-target genomic regions in the developing hypothalamus, we performed ChIP with antibodies against H3K4me1/2 and H3K27ac in E15 hypothalamus isolated from *Mll4*-cKO and littermate control mice following the treatment with AR-42 or vehicle. H3K4me1/2 and H3K27ac levels in Mll4-target loci reduced in *Mll4*-cKO mice, which was restored by AR-42 treatment (Fig. 7c). Such chromatin mark change was not observed in the negative control genomic region *Untr6* (Fig. 7c). These results suggest that AR-42 is capable of rescuing the defective chromatin landscape caused by Mll4 inactivation. Remarkably, AR-42 treatment also significantly increased the number of cells expressing key GHRH-neuronal fate markers *Ghrh*, Dlx1, and Th in E17.5 *Mll4*-cKO embryos (Fig. 7b, Supplementary Fig. 4b), indicating that deficiency of GHRH-neuronal developmental program in *Mll4*-null hypothalamus was at least partially repaired by AR-42 treatment. Collectively, these data suggest that the restored active chromatin landscapes by AR-42 led to the partial rescue of GHRH-neuronal development in *Mll4*-cKO mice.

## Discussion

**Fig. 8:**
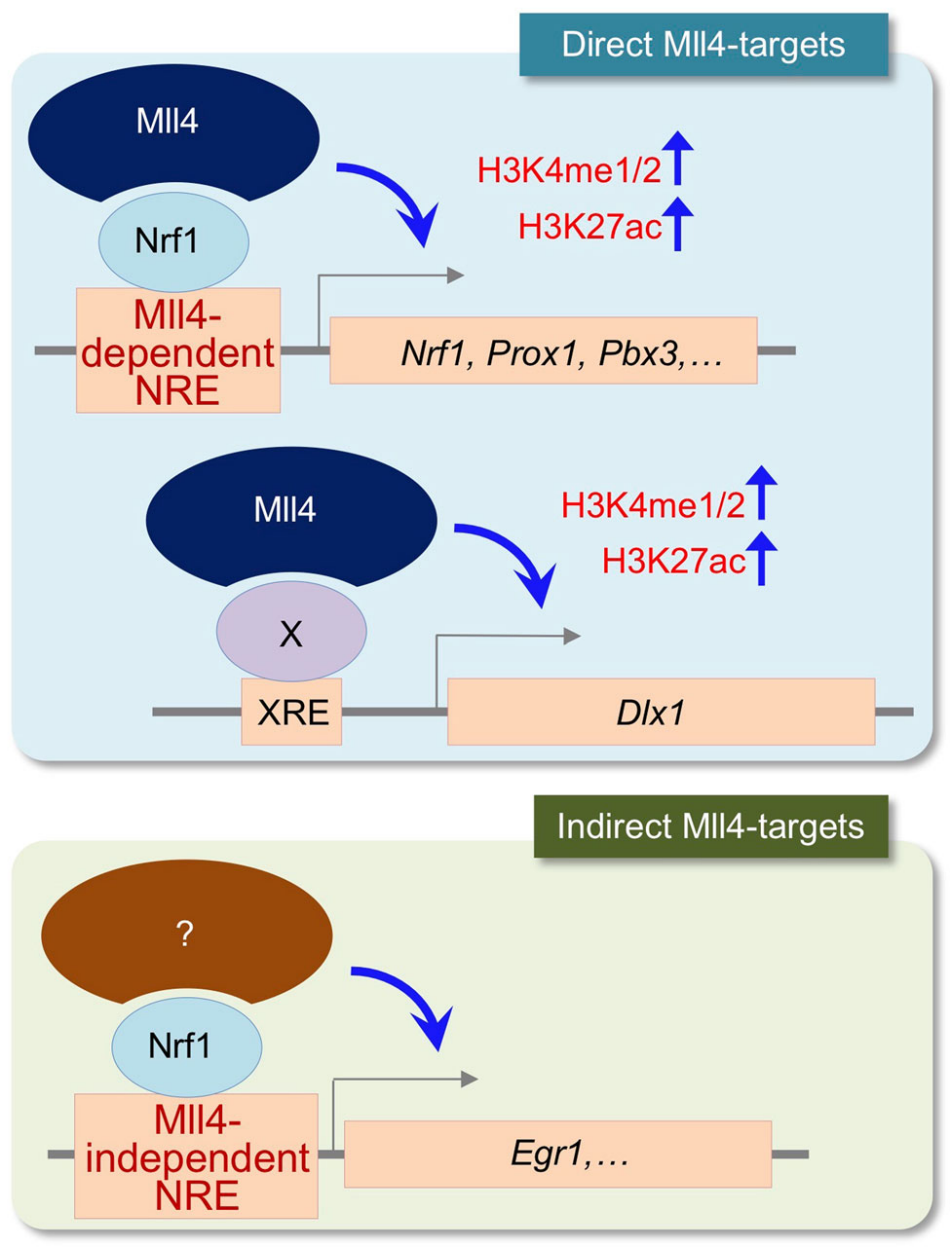
Model for Mll4 action in developing GHRH-neurons. Mll4 partners mainly with Nrf1 epigenetically activating the common target genes of Mll4 and Nrf1 by enhancing the levels of H3K4me1/2 and H3K27ac. These common targets also include the *Nrf1* gene itself, resulting in indirect activation of Mll4-independent target genes of Nrf1 by Mll4 (i.e., indirect target genes of Mll4). Mll4 also partners with unknown transcription factors, for instance to activate *Dlx1*.

Mutations in *MLL4* result in human developmental disorder KS, whose hallmarks include dwarfism^1^. Providing important insights into the etiology for the dwarfism in KS, our studies uncovered that Mll4 governs the development of mouse GHRH-neurons during hypothalamic development by establishing transcriptionally active chromatin landscapes in collaboration with Nrf1 and other partner transcription factors (Fig. 8). First, the two distinct mouse models, *Mll4*^*+/−*^ mice, which mimic the haploinsufficiency for *MLL4* in KS, and *Mll4*-cKO mice, in which *Mll4* was inactivated in the developing hypothalamus, showed impaired GHRH-neuronal development, reduced hepatic *Igf1* levels, and stunted growth. Second, our comprehensive genome-wide ChIP-seq studies for Mll4 and Nrf1 uncovered that Mll4 partners mainly with Nrf1 to activate the expression of GHRH-neuronal genes in the developing hypothalamus. Notably, Mll4, and Nrf1 collaborate to trigger the expression of the *Nrf1* gene itself, suggesting that Mll4 indirectly controls Nrf1 alone-occupied genes via increasing Nrf1 levels (Fig. 8). Our studies also suggest that Mll4 may cooperate with other transcription factors. For instance, Mll4 may be recruited to the promoter/5′UTR region of *Dlx1* by an unknown partner transcription factor. Last, the HDAC inhibitor AR-42 restored the active chromatin marks, followed by a partially rescued GHRH-neuronal developmental program, in the ARC of *Mll4*-cKO mice.

*Mll4*-cKO mice showed not only a reduced number of *Ghrh-*expressing cells but also a downregulation of many genes highly enriched in developing GHRH neurons^10^, indicating that Mll4 inactivation led to impaired GHRH-neuronal development, rather than just downregulation of the *Ghrh* gene. These GHRH-neuronal genes include transcription factor genes *Dlx1*, *Egr1*, *Pbx3*, and *Prox1*, which are directly bound and controlled by Mll4 according to our ChIP-seq and subsequent analyses. Given that Dlx1 is crucial for GHRH-neuron generation and other transcription factors are candidates for key regulators of GHRH-neuronal development^10,34^, we propose that Mll4 activates the central gene regulatory network for GHRH-neuronal development, thus serving as a primary epigenetic regulator for GHRH-neuronal production (Fig. 8).

The findings that KS patients respond well to recombinant human GH in catch-up growth^5^ indicate that KS patients have an intact GH signaling pathway. These studies imply that the hypothalamus–pituitary axis producing GH is defective in KS. While our studies demonstrate an essential role of Mll4 for the generation of GHRH neurons, it is possible that Mll4 plays an additional role in GH production in the pituitary. In this regard, it is noteworthy that Nkx2-1 regulates GH and prolactin transcription in the rat pituitary^44^. Notably, Nkx2-1 is also expressed in other cell types of the hypothalamus as well as forebrain and lung^31,45,46^. Therefore, we do not exclude the possibility that the dwarfism observed with our *Mll4*-cKO mice may also involve deletion of *Mll4* in Nkx2-1^+^ non-GHRH-neuronal populations in the hypothalamus or Nkx2-1^+^ cell types in the pituitary, forebrain or lung, either directly or indirectly. In particular, further studies are needed to investigate the role of Mll4 in the development and function of the pituitary, particularly regarding the pituitary production of GH.

The hippocampal memory defects of *Mll4*^*+/−*^ mice can be rescued by the HDAC inhibitor AR-42 or a ketogenic diet that increases the endogenous HDAC inhibitor β-hydroxybutyrate^43,47^. Both methods robustly increased H3K27ac levels^43,47^. Here, we discovered that AR-42 also rescued the expression of *Ghrh* and other GHRH-neuronal genes impaired in the ARC of *Mll4*-cKO embryos. Notably, we wished to further test if the restored expression of *Ghrh* by AR-42 also reverses the postnatal stunted growth of *Mll4*-cKO mice, but AR-42-treated pups showed perinatal lethality to severe developmental deficits, consistent with the reported embryonic lethality of knockout mouse models for several *Hdac* genes^48^. Interestingly, we found that both H3K27ac and H3K4me1 increased upon AR-42 treatment in *Mll4*-cKO mice, raising the question of which enzyme contributed to the induction of H3K4me1 in the absence of Mll4. The compensatory increase in Mll3, the paralog of Mll4, in the ARC region of *Mll4*-cKO mice (Supplementary Fig. 4c) may have been responsible for the increased H3K4me1 levels following AR-42 treatment in *Mll4*-cKO mice, which warrants future studies. Given that *MLL3* mutation has not been linked to KS or dwarfism in human, MLL3 is unlikely to function redundantly with MLL4 in the hypothalamus at least under normal condition.

Our results also highlight the intimate link between the two enhancer marks H3K27ac and H3K4me1/2. MLL4 has been shown to play a role to recruit H3K27-acetyltransferases p300 and CBP to its target genes^24–26^. Combined with these previous findings, our study suggests that MLL4 orchestrates the establishment of transcriptionally active chromatin landscape. It remains to be addressed whether MLL4-directed H3K4me1/2 modification plays an active role in gene induction or simply serves as a co-occurring mark with H3K27ac. In this regard, it will be interesting to test whether the rescue of GHRH-neuronal gene expression requires a restoration of levels of H3K27ac alone or both H3K27ac and H3K4me1/2. To this end, the chemical inhibitors of the H3K4‐methyltransferase activity of Mll4 and the H3K27-acetyltransferase activity of p300 can be explored. Such inhibitors exist for p300 (ref. ^49^), but not for Mll4 yet. Overall, our results suggest that the missing epigenetic regulatory activity of Mll4 is the main driver for the loss of GHRH-neurons in *Mll4*-cKO mice, and further provide the proof-of-concept that MLL4 can be a feasible target to develop epigenetic therapeutics for KS.

In summary, by combining mouse genetics, genome-wide studies, and pharmacological approaches, we demonstrated that Mll4 directs the development of mouse GHRH-neurons via its ability to modulate H3K4me1/2 and H3K27ac levels on its target genes during hypothalamus development. We also found Nrf1 as the major partner transcription factor of Mll4 in directing the development of mouse GHRH-neurons. These results strongly suggest that the dysregulation of MLL4-directed epigenetic regulation of GHRH-neuronal genes is likely a molecular etiology underlying the dwarfism in human KS patients.

## Methods

### Mouse work

All mice were maintained on a normal 12 h light, 12 h dark cycle with ad libitum access to chow and water, unless otherwise noted. *Mll4*^*+/−*^, *Mll4*^*f/f*^, *Pomc-Gfp*, *Npy-Gfp*, and *Nkx2-1-Cre* mice have been described previously^21,27,29–31^*. Mll4*^*f/f*^ mice were crossed with *Mll4*^*f/+*^*;Nkx2-1-Cre* mice to generate *Mll4*^*f/f*^*;Nkx2-1Cre* mice (*Mll4*-cKO mice). Pregnant mice were intraperitoneally injected with AR-42 (50 mg/kg, purchased from Selleck Chemicals) or vehicle (0.5% methylcellulose, 0.1% Tween-80, water) from 9 days after detecting a vaginal plug till harvest at E12.5 or E17 for analyses. All studies were approved by the Institutional Animal Care & Use Committee of University at Buffalo and Oregon Health & Science University.

### Cell culture and luciferase assay

HEK293 cells were obtained from ATCC and cultured in DMEM supplemented with 10% heat-inactivated fetal bovine serum (FBS) (Thermo Scientific), penicillin/streptomycin, and l-glutamine (both Lonza). For luciferase assays, cells were seeded into 48-well plates and transfected with *Nrf1*-, *Vat1*-, or *Flywch2*-luciferase reporters and expression vectors for Nrf1 or Dlx1 or control shRNA or sh-Mll4 (ref. ^27^) using SuperFect (Qiagen) according to the manufacturer’s instruction. Two days after transfection, luciferase activity was measured. The actin-β-galactosidase plasmid was cotransfected for normalization of the luciferase activities to transfection efficiency. Data were shown in relative luciferase units (mean ± SEM).

### CoIP

For coIP, HEK293 cells were transfected with expression vector for Flag-Nrf1 using calcium phosphate transfection method. Transfected HEK293 cells were subsequently dissolved in RIPA buffer and rotated for 2 h at 4 °C. After centrifugation, the lysate was subjected to immunoprecipitation using 1 mg of Mll4 antibody o/n, followed by 2 h incubation with Protein A/G agarose beads (Thermo Fisher Scientific). Beads were than dissolved in SDS loading buffer and separated on SDS-PAGE and transferred to nitrocellulose membranes. The membranes were blocked in Odyssey blocking buffer, and incubated with primary antibodies against Flag-tag and incubated o/n. Next day, the blots were incubated with secondary antibodies, and fluorescence was detected with the Odyssey System (LI-COR), as shown in Fig. 4h (the uncropped original image in Supplementary Fig. 5).

### RNA isolation and quantitative RT-PCR

Total RNA was isolated with Trizol, and converted to cDNA with a RevertAid kit (Thermo Scientific). Twenty nanograms of cDNA was used for qPCR with the ViiA7 platform and primers for amplification of *Igf1* and *Gapdh*. *Igf1* mRNA expression values were normalized to *Gapdh* using the ΔΔCt method. Relative or fold expression levels were calculated from three individual experimental replicates.

### ChIP and ChIP-seq

ChIP on E15.5 hypothalamus was performed using our homemade Mll4 and Nrf1 antibodies (ref. ^27^ and Supplementary Fig. 6). Hypothalami were dissected out, crosslinked for 10 min with PFA, and then quenched by 125 mM glycine. After washing in PBS, cells were lysed with lysis buffer, followed by lysis of nuclei with nuclei lysis buffer and sonification with the Pico Biorupter to generate 200–400 bp fragments. Chromatin was then 10× diluted in ChIP dilution buffer and, for immunoclearing, incubated with IgG and protein A agarose beads for 1 h. ChIP antibodies were added for o/n incubation followed by 1 h of incubation with agarose beads. Then, the beads were washed with RIPA buffer (0.1% SDS, 1% Triton X-100, 2 mM EDTA, 20 mM Tris-HCl, pH 8.0, 150 mM NaCl), high salt buffer (same components as in TSE, except 500 mM NaCl), Buffer III (0.25 M LiCl, 1% NP-40, 1% deoxycholate, 1 mM EDTA, 10 mM Tris-HCl, pH 8.0), LICI buffer and TE, dissolved into TE supplemented with 10% SDS and proteinse K, incubated for 4 h at 37 °C, and decrosslinked at 65 °C o/n. DNA was subsequently isolated and subject to ChIP-seq^50,51^.

### ISH and IHC

Embryonic and P0 brains were removed and fixed in 4% PFA o/n, cryoprotected with sucrose gradients, and frozen in OCT blocks, followed by sectioning using a microtome with a thickness of 12 µm per section. P33 and P65 mice were intraperitoneally injected with Avertin before performing standard perfusion with PBS and 4% PFA, followed by fixation in 4% PFA o/n. ISH was performed at 68 °C overnight with indicated RNA probes. After hybridization, slices were incubated in washing buffer (50% formamide, 1× SSC solution, and 0.1% Tween20) for 1 h, blocked in MABT buffer + 4% BSA for 1 h, and incubated with an anti-digoxigenin-AP antibody (11093274910 Roche, 1:5000) in MABT buffer + 2% BSA. Next day, the color reaction was performed with NBP/BCIP after washing with MABT buffer. For subsequent co-staining of visualized RNA probes, hybridized sections were incubated with our homemade antibodies against Mll4 (ref. ^27^, 1:1000) or Dlx1^34^ (1:1500). Next day, Vectastain ABC Elite kit (PK-6101, Vector labs) was used for the color reaction. In addition to our previously described ISH probes against *Ghrh*, *Npy*, *Trh*, *Sf1*, and *Mash1*, we also generated new RNA probes for *Pik3r1*, *Resp18*, *Pbx3*, and *Plekhg1* by converting hypothalamic RNA of P0 mice to cDNA using the primers listed in Supplementary Table 1. PCR products were then digested with the indicated enzymes and ligated into pBluescript. Digoxigenin-labeled riboprobes were generated using T7 RNA polymerase followed by purification over a column.

IHC was performed by incubating brain sections with homemade antibodies against Mll3, Mll4, Isl1, Dlx1, or Gfp (Supplementary Fig. 7) and commercial antibodies against Foxp2 (Abcam 16046, 1:2000), Prox1 (AngioBio, 1:500), Th (AB152, Millipore, 1:500), H3K4me1 (Abcam, 1:2000), H3K4me2 (Abcam, 1:1500), H3K4me3 (Abcam, 1:1000), H3K27Ac (Abcam, 1:750), Nkx2-1 (Abcam, 1:1000) in blocking buffer o/n at 4 °C. Next day, slices were washed with PBST and incubated with secondary fluorescence antibodies followed by washing and counter staining with DAPI.

### Quantification and statistical analyses

For quantification of ISH/IHC images, serial sectioning was performed on embryo/mouse brain with the distance between sections 84–216 μm. One slide from each mouse that contains matched sections was used to compare controls and mutants. Zeiss Axio imager 2 with apotome was used to image ISH and IHC results. Integrated density measurement in Image J software was used to analyze densitometry. For cell counting, specifically immuno-stained cells in the arcuate nucleus were counted. Quantifications were done by analyzing three rostral to caudal sections for each embryo/mouse and at least three embryos/mice per each experimental group. Statistical differences were determined by two-sided Student’s *t*-test. Statistical significance is displayed as follows: ns for not significant, *p < 0.05, **p < 0.01, ***p < 0.001, and ****p < 0.0001.

## Supplementary information

Supplemental Information

## Data Availability

All data supporting the findings of this study are provided within the paper and its supplementary information. A source data file is provided with this paper. The ChIP-seq dataset have been deposited in GEO (GSE149439). All additional information will be made available upon reasonable request to the authors. Source data are provided with this paper.

## Source data

Source data
