# Supplementary material for: The histone H3-lysine 4-methyltransferase Mll4 regulates the development of growth hormone-releasing hormone-producing neurons in the mouse hypothalamus
Source: Nat Commun. 2021 Jan 11;12:256. doi: 10.1038/s41467-020-20511-7 (PMC7801453; doi:10.1038/s41467-020-20511-7)
Supplement: Supplementary file 1 — Supplemental Information [file 41467_2020_20511_MOESM1_ESM.pdf]

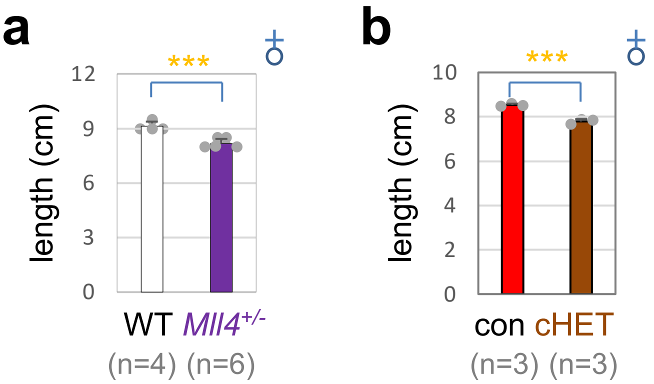

Supplementary Figure 1. Body length measurement of female *Mll4* heterozygous mice; *Mll4*$^{+/-}$ and littermate WT controls (**a**) and *Mll4*-cHET and littermate controls, both sets at P40 (**b**). The number of mice used is as indicated below each genotype in parenthesis. Statistical differences were determined by Student's t-test; p < 0.001 (three asterisks). Column bars represent mean, error bars indicate SD.

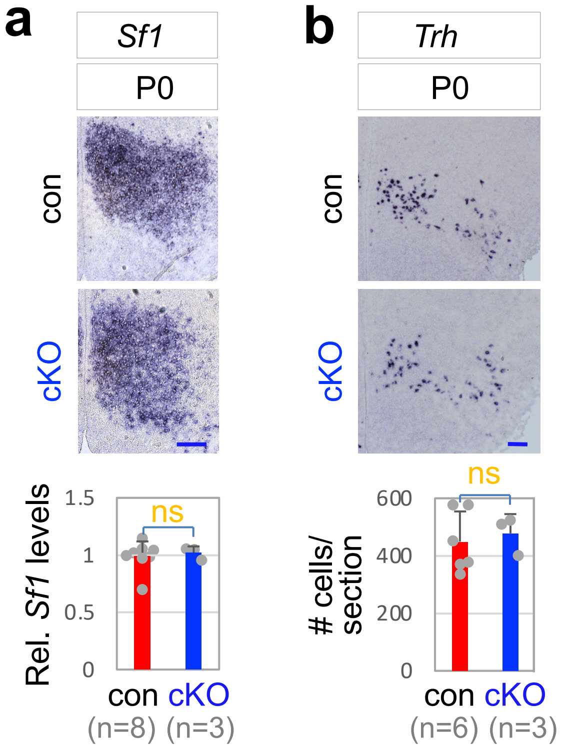

Supplementary Figure 2. Expression of *Sf1* (**a**) and *Trh* (**b**) was not significantly different between *Mll4*-cKO and littermate controls at P0. Quantifications were done by the relative intensity of signals in three rostral to caudal sections for each mouse, and the number of mice used is as indicated below each genotype in parenthesis. Scale bars, 100 μm. Statistical differences were determined by Student's t-test; not significant (ns). Column bars represent mean, error bars indicate SD.

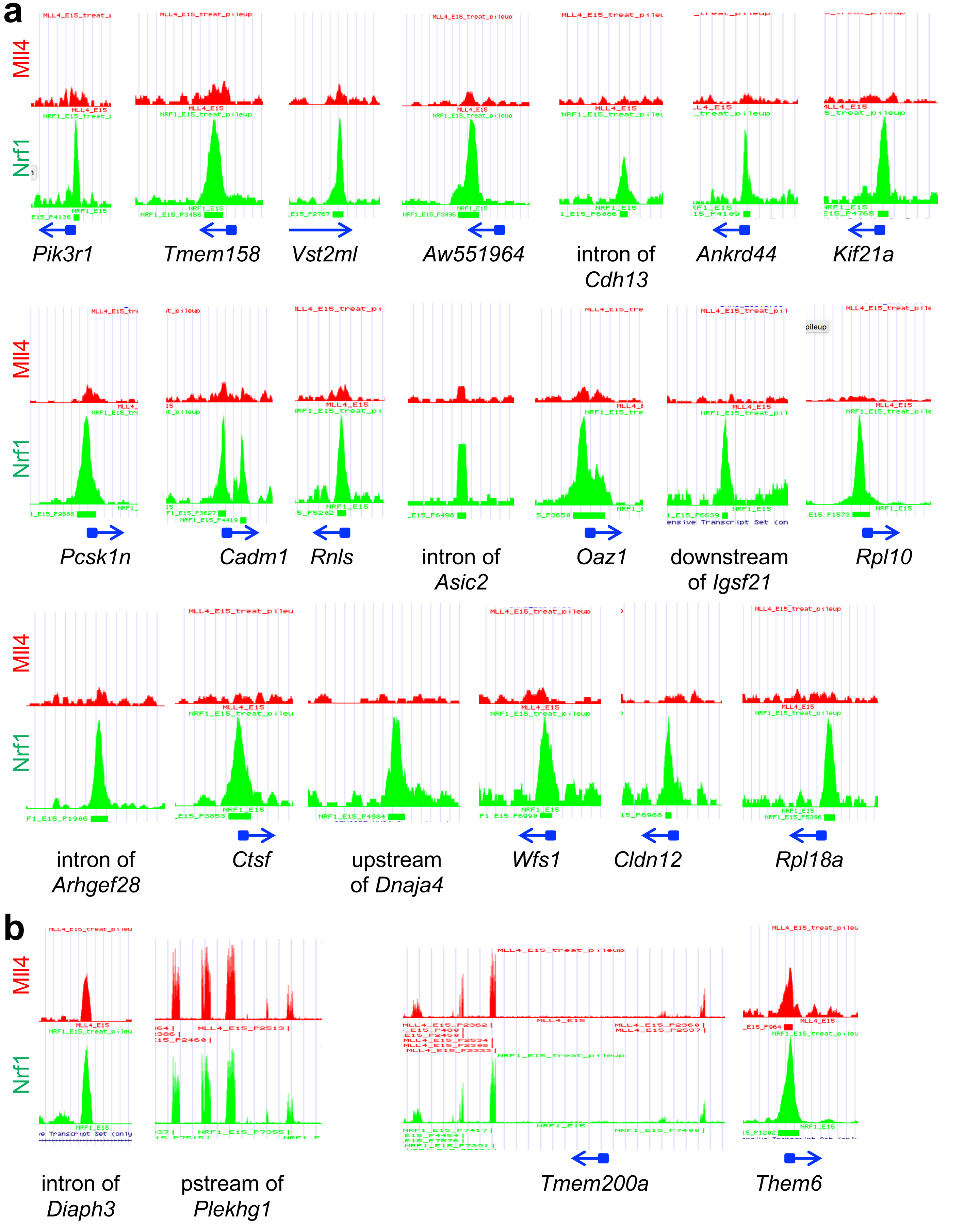

Supplementary Figure 3. ChIP-Seq peaks for Mll4 and Nrf1 in E15 hypothalamus. Genes associated with ChIP-Seq peaks for Nrf1 alone (**a**) and for both Mll4 and Nrf1 (**b**). The location of each peak with regard to its associated gene is marked by an arrow for the direction of the gene and a square for the 5' UTR (both in blue).

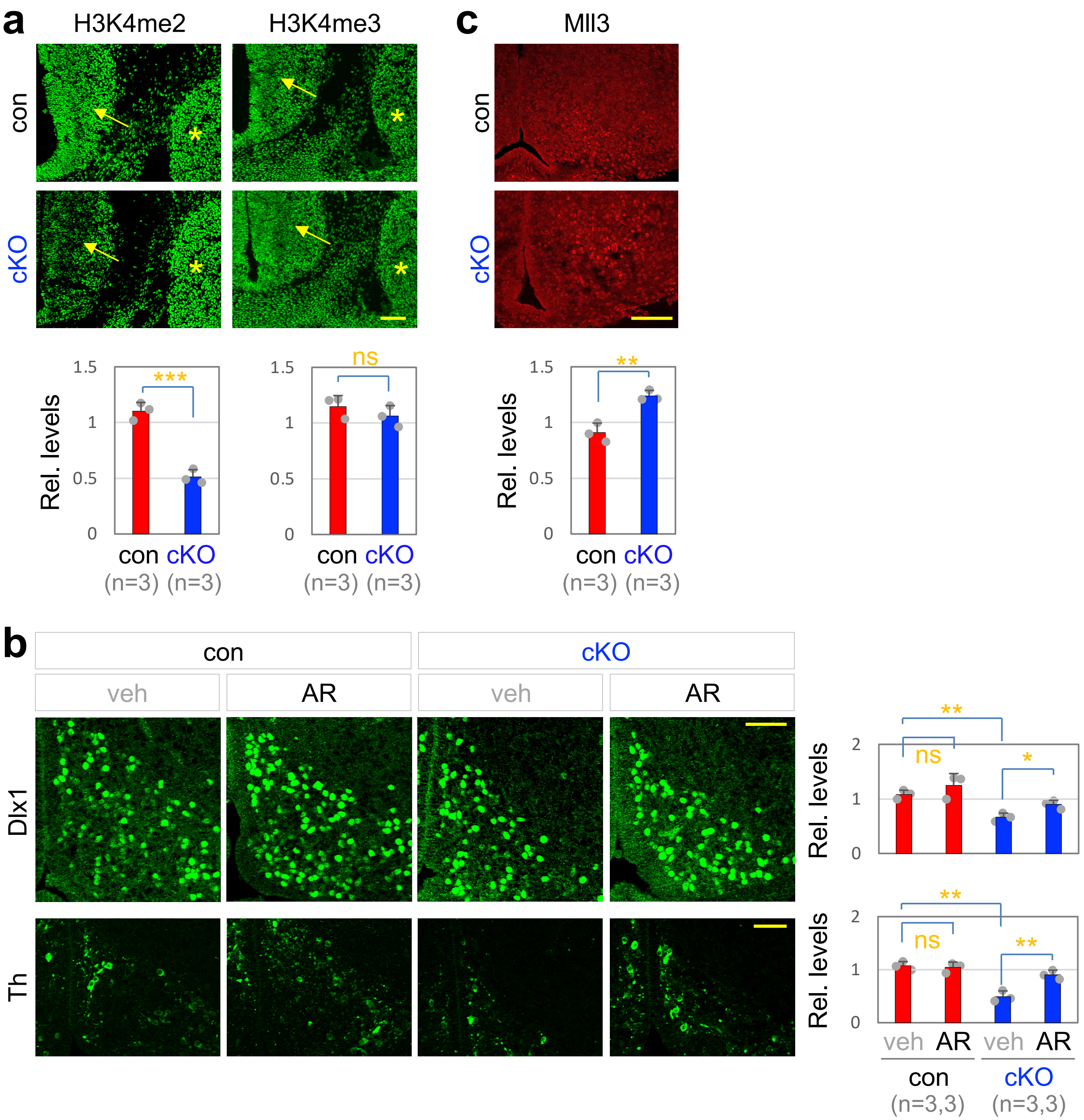

Supplementary Figure 4. a Levels of H3K4me2 but not H3K4me3 are reduced in *Mll4*-cKO animals relative to their littermate controls at E12.5. Quantification of H3K4me2/me3 levels in the developing ARC (arrows) were done relative to the signals in the adjacent tissue (the trigeminal ganglion, indicated by asterisks). **b** IHC analyses of Dlx1 and Th expression in embryos harvested at E17.5. **c** Mll3 levels are upregulated in *Mll4*-cKO animals relative to their littermate controls at E17.5. **a-c** Statistical differences were determined by Student's t-test; $p < 0.05$ (one asterisk), $p < 0.01$ (two asterisks), $p < 0.001$ (three asterisks) and not significant (ns). Column bars represent mean, error bars indicate the SD. Scale bars, 100 µm. The number of mice used is as indicated below each genotype in parenthesis (**a-c**).

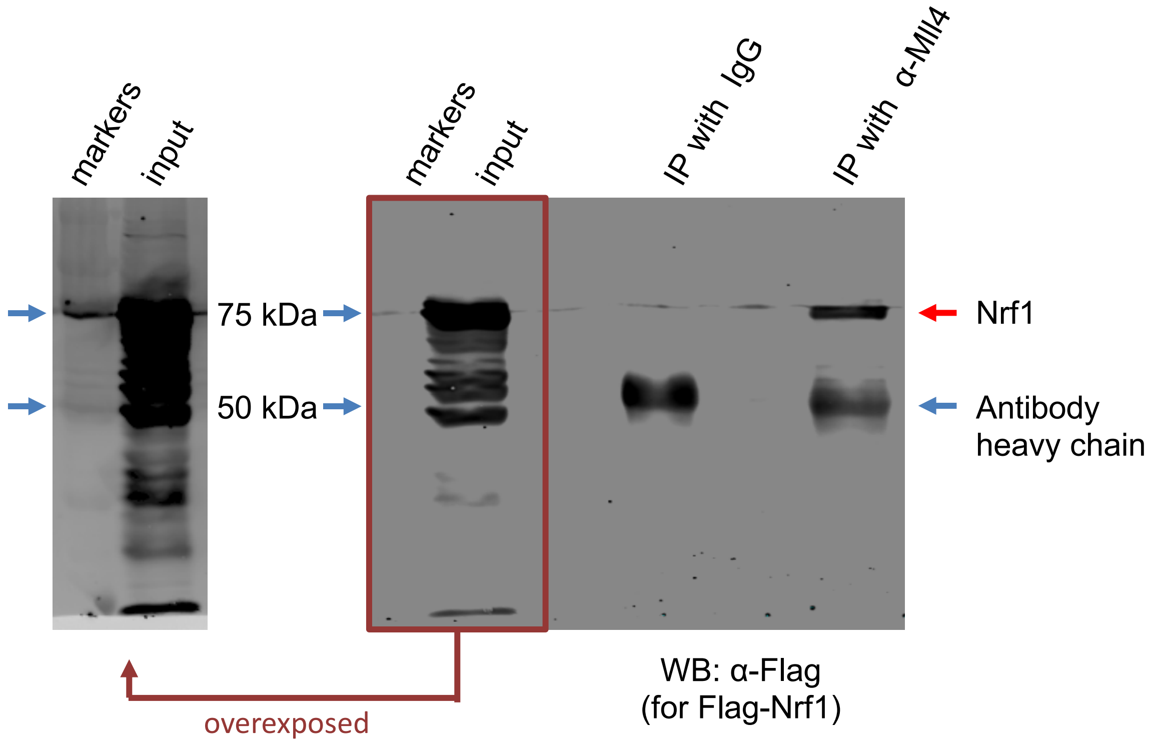

Supplementary Figure 5. Uncropped original image for the coIP experiments presented in Fig. 4h. The size marker ladder lane was clearer in overexposed image (left panel).

Control　　　*Nrf1*-cKO

Control

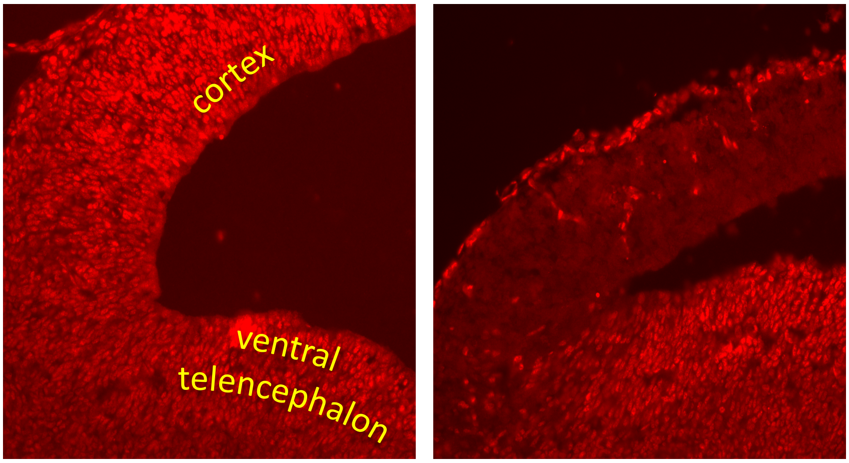

Supplementary Figure 6. Validation of our home-made anti-Nrf1 antibody for specific detection of Nrf1 protein. In immunohistochemical analysis with our home-made anti-Nrf1 antibody in the embryonic forebrain from the cortex-specific *Nrf1* conditional knockout (*Nrf1*-cKO) or littermate control mouse, Nrf1 is broadly expressed in the embryonic forebrain of the control mouse. In contrast, in *Nrf1*-cKO mouse, Nrf1 was detected in the ventral telencephalon, but not in the cortex in which *Nrf1* is conditionally eliminated.

Supplementary Table 1: Primers used in this study

1) Primers for ISH
Pik3r1-forward, tataagcttggcgtgacatgtaggctctcag
Pik3r1-reverse, tatctcgagaaaggtcccatcagcagtgtc
Resp18-forward, gagaattccgctagagggtgaaaagtgac
Resp18-reverse, tataagcttggcctttgggattactttggtg
Pbx3-forward, ggaattcaagggtcccaagtcggagcc
Pbx3-reverse, accaagcttttgacaagtctgtgcagcacctag
Plekhg1-forward, tagaattcatgccacacctgtctaactccttg
Plekhg1-reverse, attaagcttcttcttgcagtgagccttctacac

2) Primers to detect Igf1 in the liver
Igf1-forward, tcatgtcgtcttcacacctct
Igf1-reverse, tccacaatgcctgtctgagg

3) Primers for validation ChIP
Dlx1/2-forward, tacccctacgtcaactcggt
Dlx1/2-reverse, cgcacgtacctgggtcct
Resp18-forward, atctccacccgcagtgtttt
Resp18-reverse, cagaatacctgcgcagacca
Egr1-forward, cccttagggatgggactggg
Egr1-reverse, atccaaaacaaacgtcctgcc
Prox1-forward, atgttcggaccttgcctcta
Prox1-reverse, tcacgtgagagagggcacaa
Vat1-forward, gcttctcaaagctccctcgg
Vat1-reverse, ctgcccactaaacactcccg
Flywch2-forward, acctcaagctagggtcacgg
Flywch2-reverse, atcgagcgttcaacttgtcg
Pbx3-forward, gttaacctgctccagaaacgg
Pbx3-reverse, gaacgagaagtcgctgcctc

4) Primers for H3K4me1/H3K27ac ChIP
Nrf1-forward, tctctcccacatctcccagg
Nrf1-reverse, cagcaacgacacaacaggtg
Vat1-forward, tccgcggacatggctaga
Vat1-reverse, tatcagtcacacgcacgtaca
Flywch2-forward, cacctcaagctagggtcacg
Flywch2-reverse, ccaggagggagtcagagtca

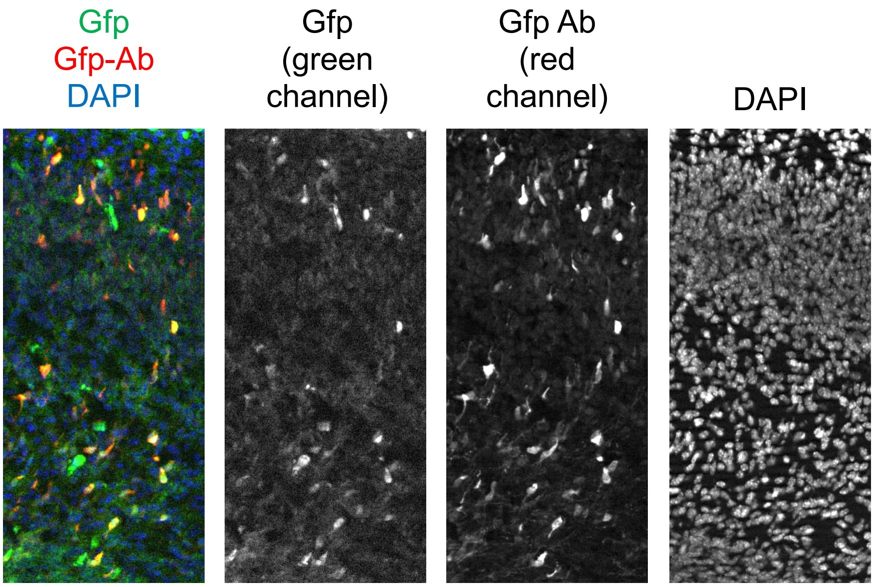

Supplementary Figure 7. Validation of our home-made anti-Gfp antibody for specific detection of Gfp expression. The Gfp-expression plasmid was introduced to the cells in the embryonic cortex using in utero electroporation. The cortex was collected three days after electroporation and immunostained with anti-Gfp antibody. The green signal shows the detection of Gfp expression without any antibody. The red signal represents the detection of Gfp expression using our home-made anti-Gfp antibody. The anti-Gfp antibody intensified the weak Gfp signal, but produced no signal in cells that do not express Gfp.
